# Effective Computation of Coupling Force Constants: Metal Carbonyls as a Test Case

**DOI:** 10.1021/acs.jctc.5c00645

**Published:** 2025-09-11

**Authors:** Henrik Borgman, Somi Vasisth, Jörg Grunenberg

**Affiliations:** Institute of Organic Chemistry, 26527 TU Braunschweig, Hagenring 30, 38106 Braunschweig, Germany

## Abstract

An automated protocol enabling the efficient computation of unique potential coupling constants is presented. Several modern density functional (DFT) methods are tested against coupled cluster theory (CCSD­(T)) in order to evaluate their quality in producing reliable compliance matrix off-diagonal elements. While force coupling constants could serve as descriptors of electron delocalization in general, we tested the ability of coupling compliance constants as descriptors of the Dewar–Chatt–Duncanson model in VCO^–^, CrCO, MnCO^+^, FeCO^2+^, NiCO, CuCO^+^, FeCO^+^ and the isoelectronic hexacarbonyls of the 3d and 5d series from Ti to Co, and Hf to Ir, respectively. A robust semiautomated algorithm including the computation of all compliance coupling constants as inverse covariant second derivatives is implemented in our open source version of the COMPLIANCE code.

## Graphical abstract

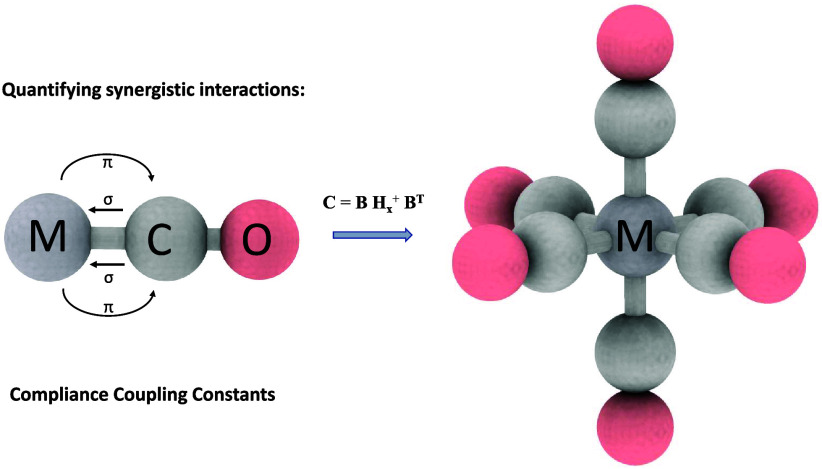

## Introduction

The traditional information flow concerning vibrational spectroscopy begins with the recording of an experimental IR (or Raman) spectrum, followed by a back calculation of the individual force constants applying Wilson’s normal-mode analysis.^1^ The opposite direction, that means, starting with the knowledge of all force (and coupling) constants followed by the prediction of vibrational spectra from these data, was the dream of the early spectroscopists.^2^ This dream was hampered by the fact that, apart from several stretch/stretch coupling constants,^3^ most of these coupling data were not known at that time.

With the unprecedented developments in modern software and hardware technology this dream seems to have come true in the last two decades,^4,5^ especially since the development of codes that allow the automatic computation of compliance constants (“relaxed force constants”) or local force constants routinely.^6–27^

The use of compliance constants is nearly as old as vibrational spectroscopy itself. In 1947 Pitzer and Taylor^28^ mentioned compliance matrices **C** as the inverse of the force constant matrix **F** ^–1^ = **C**. At another step in 1953 Decius^29^ recognized that individual force constants strongly depend on the definition of all other coordinates while, in contrast, the inverted matrix of force constants **C** does not.

The reason for this difference and the physical significance of **C** is the following: just as the potential energy can be written as a quadratic form in the internal coordinates, it can also be written as a quadratic form in terms of generalized forces, which are allowed to relax. The corresponding proportionality constants are the elements of the compliance matrix **C**.

The idea of compliance constants (or relaxed force constants, the reciprocals of the diagonal terms in **C**) seems to disappear and arise again in the chemical literature after these initial, limited explorations: In the late 1960 Jones^30^ and Machida^31^ borrowed the concept again. 1977 Dewar and Swanson used compliance constants in order to study carbonyl complexes,^32^ while in the early 1980s I. H. Williams proposed compliance constants as an ideal descriptor of bond strengths in organic chemistry.

Starting in 2000 ^33^ we proposed the use of *generalized* force constants for interactions of both covalent^34^ and noncovalent^35^ nature, the latter including agostic^36^ and C–H···Anion interactions^37^ as well as interstitial carbon atoms.^38^ We further developed an open source code, which is able to compute all diagonal *and coupling terms* of the compliance matrix. In 2016 Schaefer III et al. again proposed relaxed force constants as descriptors for hydrogen bond strengths,^39^ while in 2022 Manogaran and Manogaran discussed the inclusion of anharmonicity in compliance constants.^40^

Finally, in a recent remarkable publication Gernot Frenking and colleagues from the Nanjing Tech University come to the conclusion^41^ that *“the force constant is the most general measure for determining the strength of a chemical bond”*.

The authors enthusiastically recommend the use of local force constants developed by Cremer and Konkoli in 1998.^42^ Interestingly, Frenking and co-workers did not discuss relaxed force and compliance constants even though it was shown by Cremer that his local-mode force constants are identical with our relaxed force constants^43^ proposed by Decius half a century in advance (see above). Most importantly, in Cremer’s approach all coupling terms disappear.

With this publication we try to go one step further and find out if the computation of compliance *coupling* constants are of use as descriptors in chemistry.

### Theory of Coupling Compliance Constants

Within the scope of the harmonic approximation, the Taylor series expanding the potential energy of a molecule can be terminated after the quadratic term, i.e., the change in the potential energy V due to an (infinitesimal) distortion of the molecule is given by

1 $$V=G_{x}\mathrm{dX}+1/2\mathrm{dX}H_{x}\mathrm{dX}$$

where **X** denotes the column vector of Cartesian displacement coordinates, **G**_***x***_ and **H**_***x***_ are the Cartesian gradient vector and the Cartesian Hessian matrix *including all coupling force constants* by definition. Due to the rank deficiency, the Cartesian Hessian **H**_***x***_ itself does not possess an ordinary inverse producing our sought after compliance matrix.

As chemists, we are nevertheless interested in internal coordinates, which means that we are looking for the corresponding energy function with respect to a set of (nonredundant) 3N-6 internal displacement coordinates **Z**

2 $$V=G_{z}\mathrm{dZ}+\mathrm{1/2}\mathrm{dZ}H_{z}\mathrm{dZ}$$

The transformation of both Hessian matrices (Cartesian and internal) is done by Wilson’s famous **B** matrix, which maps the Cartesian Hessian to the internal subspace.

3 $$H_{x}=B^{T}H_{z}B$$

Alas, due to the fact that Wilson’s **B** matrix is rectangular, the above expression is not invertible, which means we cannot use expression (4) in order to produce our internal Hessian matrix including all coupling force constants nor the compliance matrix thereof.

Nevertheless, **B** has full row rank, and thus its transpose **B** ^T^ has a left inverse (**BuB** ^T^)^−1^ **B u**, where **u** is an arbitrary nonsingular matrix. The left inverse of **B** ^T^ is then nothing else but the famous (transposed) Moore–Penrose inverse **B** ^**+**^ of **B**,^44,45^

4 $$B^{+T}={\left({\mathrm{BB}}^{T}\right)}^{-1}B$$

which finally allows the unique transformation of the Cartesian Hessian **H** *_**x**_*, which is the output of every standard quantum chemical frequency calculation, into a (nonredundant) internal Hessian **H** *_**z**_*

5 $$H_{z}=B^{+T}H_{x}B^{+}$$

The generation of nonredundant sets of internal coordinates is not always trivial. In view of an automatization, we therefore implemented the use of redundant sets of internal coordinates. As stated above, a Hessian in redundant internal coordinates does not possess an ordinary inverse. However, again the Moore–Penrose inverse can be used as an equivalent, leading to

6 $$C={\left(B^{+T}H_{x}B^{+}\right)}^{+}$$

Employing eq 6 directly would nevertheless again need the costly evaluation of the Moore–Penrose inverse of our **B** matrix. Applying the reverse order law for Moore–Penrose inverses twice we finally obtain

7 $$C=BH_{x}+B^{T}$$

This means that the most time-consuming step of our protocol is the Moore-Penrose inversion of the Cartesian Hessian matrix, which is a simple 3N × 3N problem. Further, our algorithm now allows for the direct computation of distinct elements of **C** by choosing **B** to consist of just one row, which in turn allows us the successive construction of the compliance matrix, step by step. Again, all coupling compliance constants are included and their numerical value does not depend on the completeness or the definition of the coordinate system in general.

The above algorithm has been implemented in our open source code COMPLIANCE. Once the Cartesian Hessian is read in, and the rotatorial contributions have automatically been removed, the Moore–Penrose inverse of the Hessian is calculated by performing a standard singular value decomposition. All subsequent multiplications can utilize the sparsity of the **B**-matrices.

The program uses a graphical interface for an intuitive interaction. Reading in the Cartesian gradient and force constant matrix from a preceding quantum (or classical) chemical frequency calculation and defining the internal coordinates of interest by simple mouse clicks, the user can successively build up the compliance matrix *including all coupling constants*. Additionally, all coupling constants of a single bond can be visualized applying an external force to this very coordinate, giving rise to a specific mode, which we would like to term COMPLIANCE mode.

### Simple Organic Molecules

As a first check of our hypothesis that we might use compliance coupling constants as unique descriptors for electronic coupling, we tried to reproduce the decreasing electron delocalization between the carbon backbone in prototypic organic molecules like benzene (**1**), the allylic cation (**2**) and propene (**3**) quantitatively, applying CCSD­(T)/cc-pvtz coupling compliance constant *C_ij_* between adjacent carbon–carbon bonds.

Due to our computed compliance matrices (details in Supporting Information, SI) the coupling constants in general are lower by 2 orders of magnitude in comparison with the diagonal constants. This is in line with earlier predictions by Swanson that in compliance matrices, the coupling terms are unique and reflect a minimum value since they describe the minimum energy path (MEP).^46^ The values are small but may not be neglected as in the case of Cremer’s local mode ansatz.

The results are summarized in Figure 1. Even in benzene (**1**), a prototype of electron delocalization, the computed coupling constant *C_ij_* is only −2.31 × 10^–2^ cm/N. The negative sign mirrors the strong synergistic effect: if one c-c-bond is compressed, the adjacent c-c-bond will be elongated. As expected, going to the allylic cation (**2**), the coupling constant *C_ij_* – while still negative – is going down by ∼50% to −1.02 × 10^–2^ cm/N. In propene (**3**), with even less delocalization, we see another 50% decrease to −0.52 × 10^–2^ cm/N.

**Figure 1:**
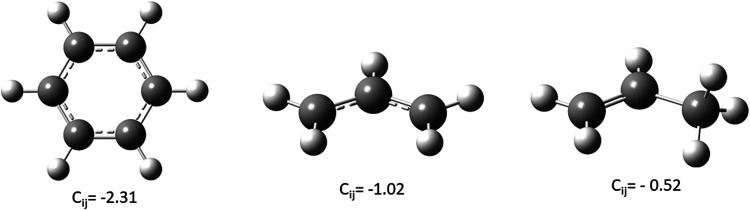
CCSD­(T)/cc-pvtz coupling compliance constant *C_ij_* in [cm/N × 10^–2^] between adjacent C–C bonds in benzene (maximal delocalization, left), the allyl cation (strongly delocalized; middle) and propene (weak delocalization, right).

With this confirmation that coupling compliance constants indeed could describe the interaction and delocalization of electrons, more complex systems were chosen in a second step.

### Transition Metal Monocarbonyls

Transition metal complexes represent a large family of complexes with highly variable metal–carbon bonds, which still represent an active challenge in chemistry, especially when it comes to transition metal carbonyl cations TMCCs.^47,48^

In order to check if modern DFT methods are generally able to reproduce the coupling between the metal atom and the carbon monoxide ligand, six different monocarbonyls (VCO^–^, CrCO, MnCO^+^, FeCO^2+^, NiCO, CuCO^+^) were studied in combination with various basis sets and density functionals. Figure 2 depicts the definition of the coordinates and calculated constants.

**Figure 2:**
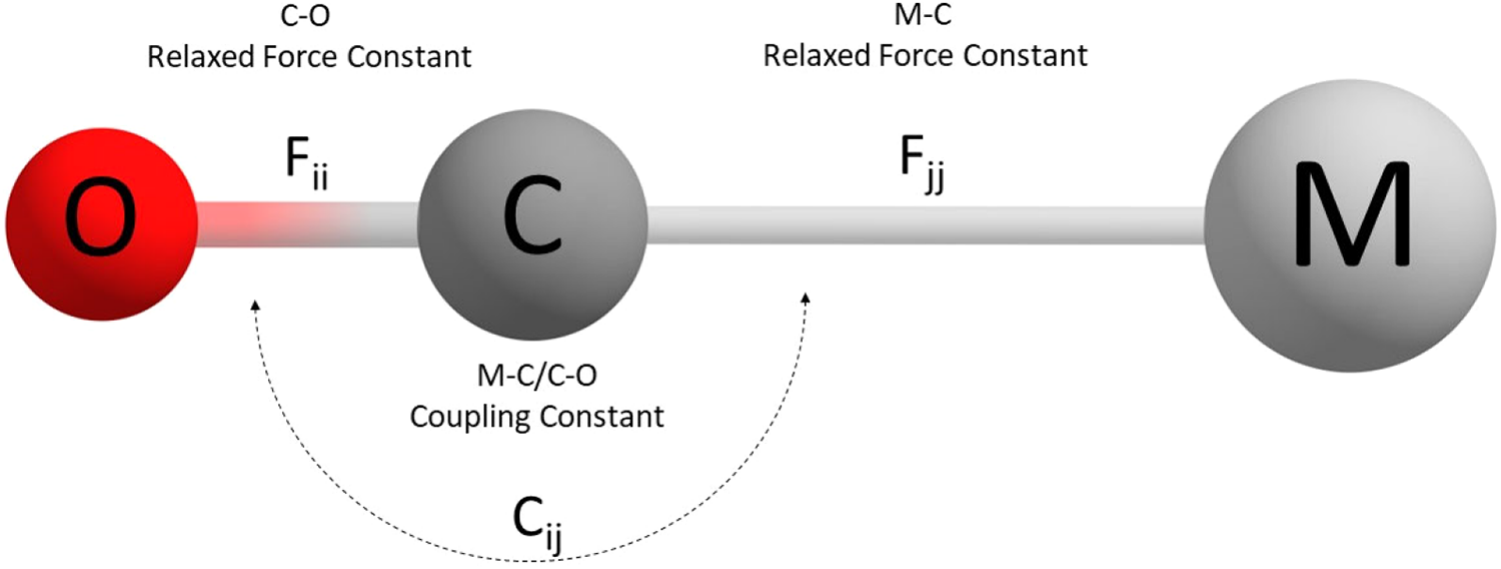
Definition of the coordinates and calculated constants for the monocarbonyl complexes in our method evaluation.

The def2-TZVPD basis set was chosen as a compromise between accuracy and efficiency. Density functionals include the historically important pure (BP86 ^49,50^ ) or hybrid (B3LYP^51,52^ ) density functionals, which are still used today, together with modern functionals including nonseparable gradient approximations (NGA) from the Truhlar group like MN15,^53,53^ nonempirical τ-dependent gradient-corrected correlation methods developed by Perdew and Scuseria (TPSSh^54^), long-range corrected hybrid density functionals from the Head-Gordon group as in ωB97X-D,^55^ as well as double hybrid functionals like Grimme’s B2PLYPD3.^56^

As a reference we chose the gold standard in single reference quantum chemistry, coupled cluster theory with single and double excitations CCSD­(T)^57^ in combination with Dunning’s correlation consistent triple-ζ basis aug-cc-pvtz.^58^

In order to get a feeling concerning the numerical values, Table 1 shows the CCSD­(T) results for two monocarbonyls the electron rich VCO^–^ and the electron poor FeCO^2+^, respectively. In case of a more or less missing pi backbonding (coupling constant: −0.001 cm/N) in FeCO^2+^ the relaxed Fe–C force constant reaches only 1.60 N/cm, together with a more or less unperturbed CO carbonyl bond (20.83 N/cm), while the pronounced backbonding in VCO^–^ (coupling constant: −0.026 cm/N) is mirrored by a doubling of the metal–carbon bond strength to 3.10 N/cm with the CO carbonyl bond being proportionally weakened (11.76 N/cm).

**Table 1:**
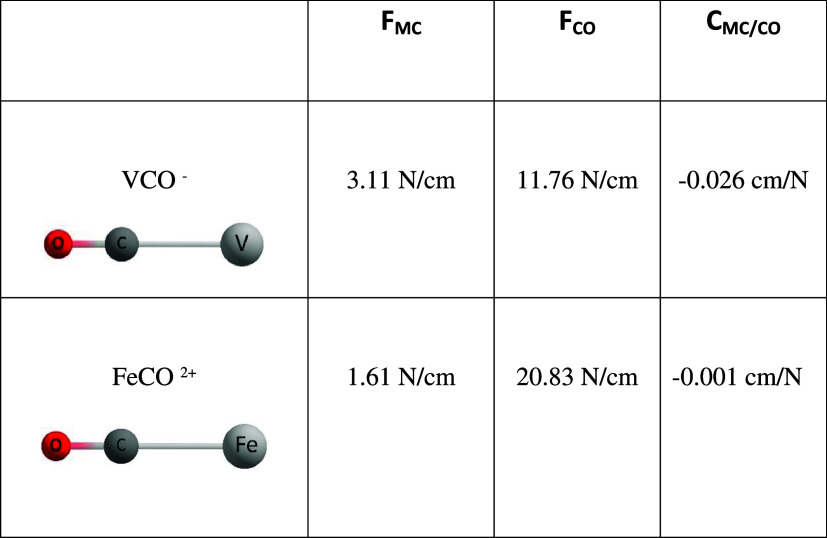
Relaxed Force Constants (Inverse Compliance Diagonal Terms) and Coupling Terms of the Compliance Matrix for VCO^–^ and FeCO^2+^ Computed at the CCSD­(T) Level of Theory^a^

a The basis set used was aug-cc-pvtz.

Even if our database seems to be limited, in the following we are trying to distill some trends for the CO, the MC and the coupling coordinates. Our CCSD­(T)/DFT benchmark results for all the subvalent monocarbonyls concerning the C–O coordinate is shown in Figure 3. We again follow the suggestion by Jones^59^ and use the inverse of the individual *diagonal* elements of the compliance matrix (“relaxed force constants”) in order to facilitate the comparison with older literature values.

**Figure 3:**
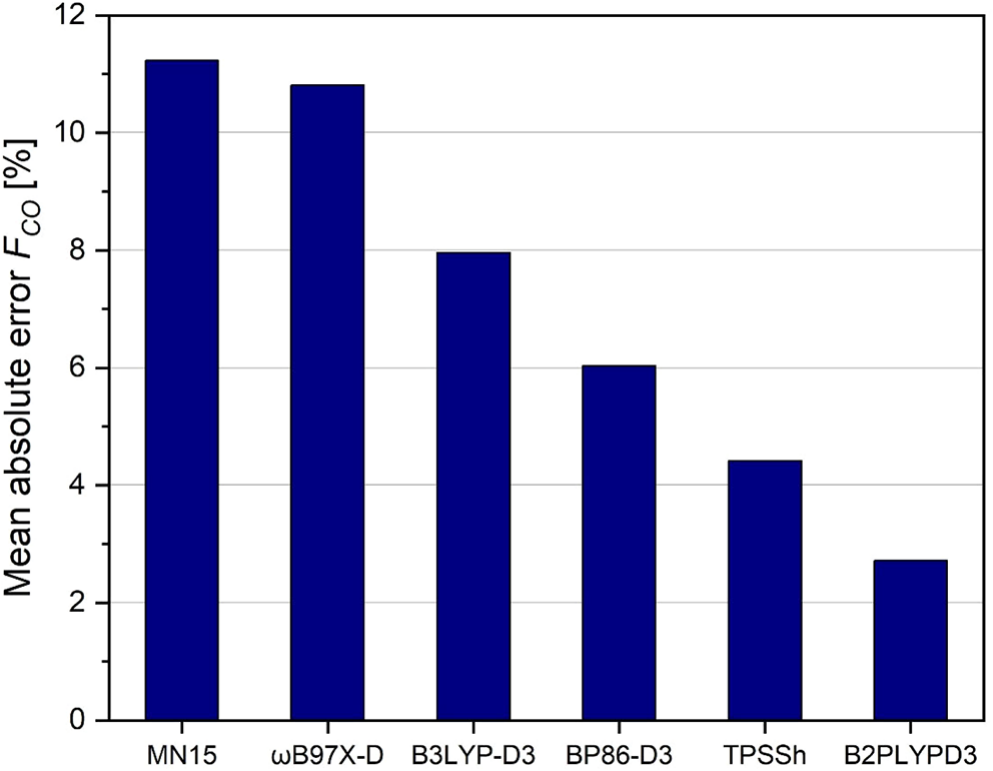
Mean absolute error of relaxed force constants (inverse compliance diagonal terms) corresponding to C–O bonds in transition metal monocarbonyls, from selected density functionals with the basis set def2-TZVPD against CCSD­(T)/aug-cc-pvtz standard.

Due to the high numerical values of relaxed carbonyl C–O force constants, the studied density functionals reproduce the CCSD­(T) values moderately well, with a mean absolute error markedly below 15%. While the NGA functionals like MN15 seem to systematically overestimate the C–O bond stiffness, independent of the electronic situation (strong backbonding in VCO^–^ versus missing backbonding in FeCO^2+^; see Table S12 in our SI), both, the pure functional BP86 as well as, to a lesser extent, the double hybrid functional B2PLYPD3 underestimate the stiffness of the C–O bond, systematically. Looking at signed mean error in the SI (Table S12) the hybrid meta-GGA functional TPSSh seems to reproduce the coupled cluster results for the C–O potential perfectly. In this case, the low signed error is the result of a cancelation between a pronounced underestimation in FeCO^2+^ and CrCO and a moderate overestimation for all the other monocarbonyls (see SI, Table S12).

Turning to the metal–carbon bonds, we see an overestimation of the M-C bond stiffness using DFT methods for nearly all monocarbonyl systems (see SI, Table S10). The overall absolute error is well below 20% for TPSSh, ωB97X-D, and the double hybrid B2PLYPD3, while MN15 and B3LYP-D3 perform even better. The overestimation of the M-C bond strength by the pure BP86 functional is obvious and explainable by the delicate synergistic interplay between donation and backbonding when it comes to the description of this very bond (see Figure 4).

**Figure 4:**
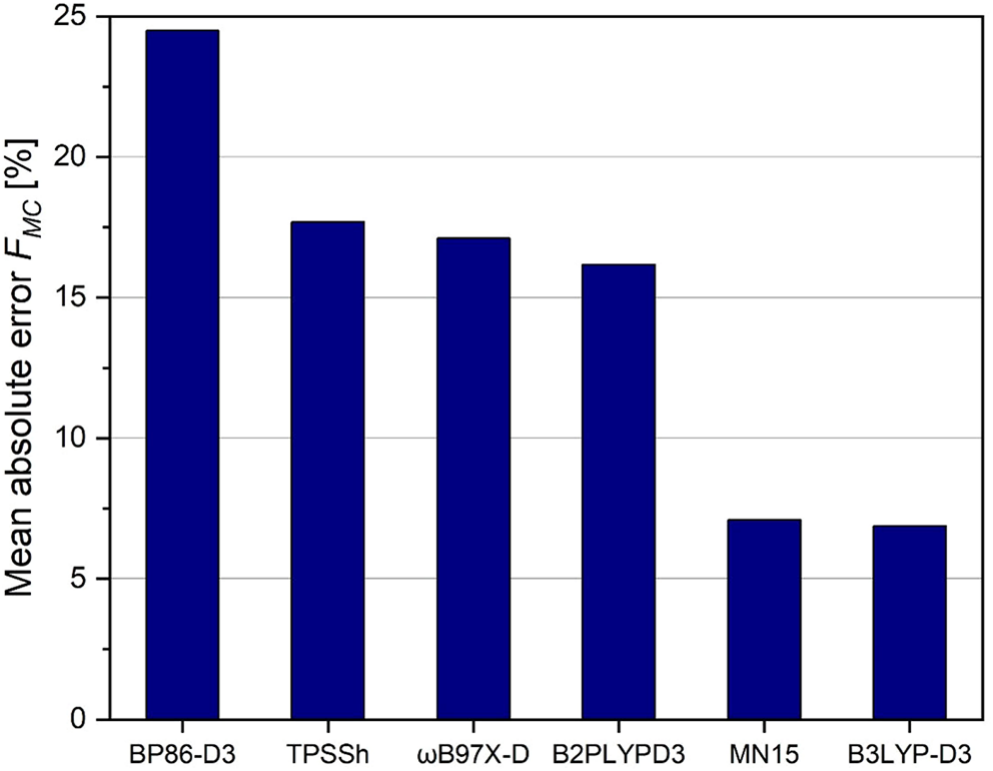
Mean absolute error of relaxed force constants, corresponding to M-C bonds in transition metal monocarbonyls, from selected density functionals with the basis set def2-TZVPD against CCSD­(T)/aug-cc-pvtz standard.

The situation is even more complicated, turning to our actual property of interest, the *compliance coupling constants*. In order to reproduce the coupled cluster results, the description of the electron correlation inherent in the synergistic sigma σ and π bonding becomes even more important. The DFT results are nevertheless quite encouraging. The absolute error in reproducing the CCSD­(T) compliance MC/CO *coupling constants*, applying modern DFT methods like MN15 is still around 30%. Again, the pure density functional BP86 seems to step out of line concerning the overestimation. In line with a study by Martin Head-Gordon et al.^60^ the range-separated functional ωB97X-D shows the best performance in reproducing the CCSD­(T) coupling *compliance constants* in our selected monocarbonyl (see Figure 5 and SI, Tables S13, S14).

**Figure 5:**
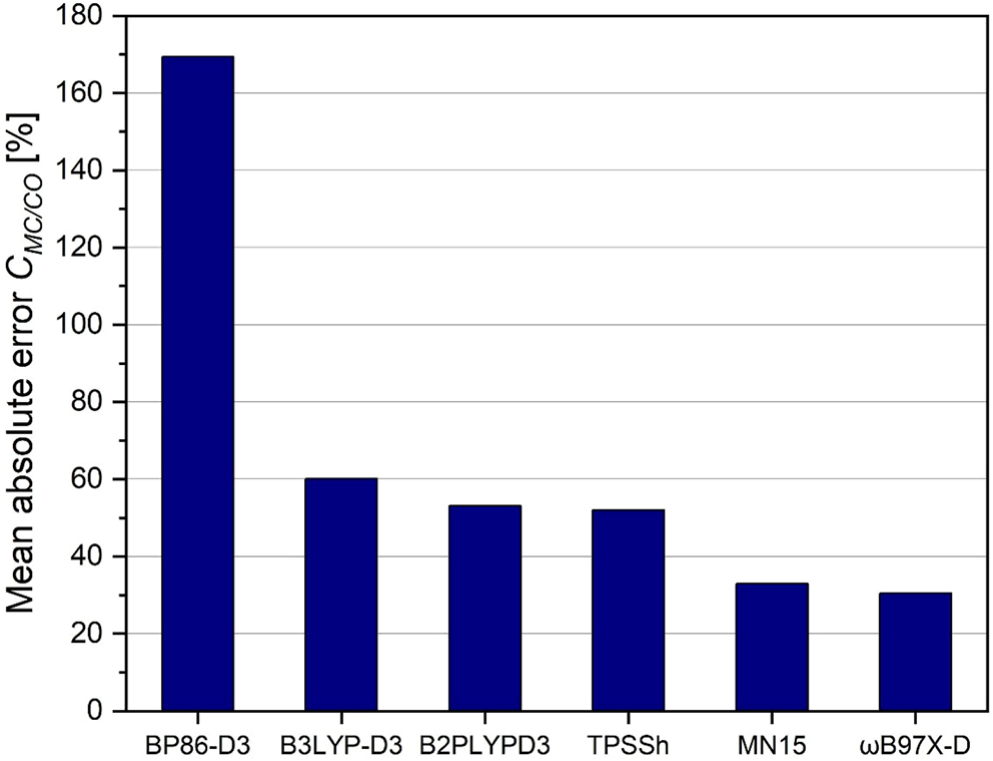
Mean absolute error of *coupling compliance constants*, corresponding to the coupling between M-C and C–O bonds in transition metal monocarbonyls, from selected density functionals with the basis set def2-TZVPD against the CCSD­(T)/aug-cc-pvtz gold standard.

The results of all tested DFT methods nevertheless strongly depends on the studied monocarbonyl system. For electron rich systems like VCO^–^, we see a pronounced overestimation of the coupling constant around 15% (−0.026 cm/N at the CCSD­(T) versus −0.030 cm/N at the MN15 level of theory; see S13 and S14 in our SI). In more or less all other systems our DFT methods seem to underestimate the coupling. We refrain from a deeper discussion of systems which lack backbonding like CuCO^+^ or FeCO^2+^, since the numerical values of the relevant coupling constants are around zero.

Even if our database is still limited, several general statements concerning the efficacy of density functionals predicting coupling force constants can be made: BP86 (in our study the only representative for a functional without HF exchange) predicts the M-C bonds to be too rigid, and the C–O bonds too soft. This seems to be the result of an overestimation of π-back-bonding, visible as a pronounced overestimation of the predicted compliance *coupling constants* between those two bonds (Figure 5). The same pattern is observed to a somewhat lesser extent for the double hybrid functional, while the traditional B3LYP slightly overestimates the rigidity of both bond types.

All in all, we advocate second generation functionals like MN15, ωB97X-D or TPSSh when it comes to the computation of moderately and systematically accurate potential constants in carbonyl complexes in general.

### Hexacarbonyls

In another check of our protocol the hexacarbonyl Cr­(CO)_6_ was chosen alongside isoelectronic, charged complexes from Ti­(CO)_6_^2–^ to Co­(CO)_6_^3+^. Additionally, the respective 5d analogues were used as a point of comparison to observe possible differences between periodic trends. Figure 6 serves to illustrate the coordinates used for the octahedral complexes. Again, the compliance *coupling constant C*_MC/CO_, in those isoelectric hexacarbonyls formed the main interest of our study.

**Figure 6:**
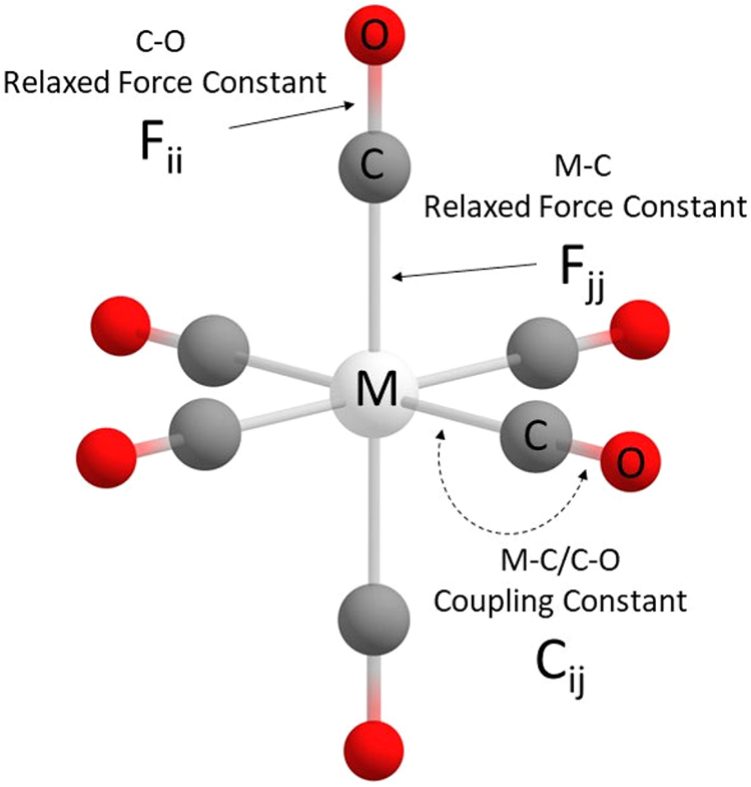
Example of the geometry of transition metal hexacarbonyls with the two analyzable bonds shown.

As expected the double negatively charged hexacarbonyls Ti­(CO)_6_^2–^ and Hf­(CO)_6_^2–^ show a pronounced **C** coupling of around −2.5 cm/N × 10^–2^ and −2.25 cm/N × 10^–2^ (TPSSh values), respectively (Figure 7). Note again the negative (synergistic) sign of the coupling constants. Both series, the 3d and the 5d hexacarbonyls, show a pronounces decrease in our computed **C**_MC/CO_ coupling constants, reflecting the expected strong and systematic decrease of the π-back-bonding going from a total charge of −2 to +3 (Figure 8). Our results reproduce the results of a recent study by Bickelhaupt et al.^61^ Our method does not try to separate the sigma donation and the pi backdonation as isolated entities but rather a quantification of the synergistic coupling.

**Figure 7:**
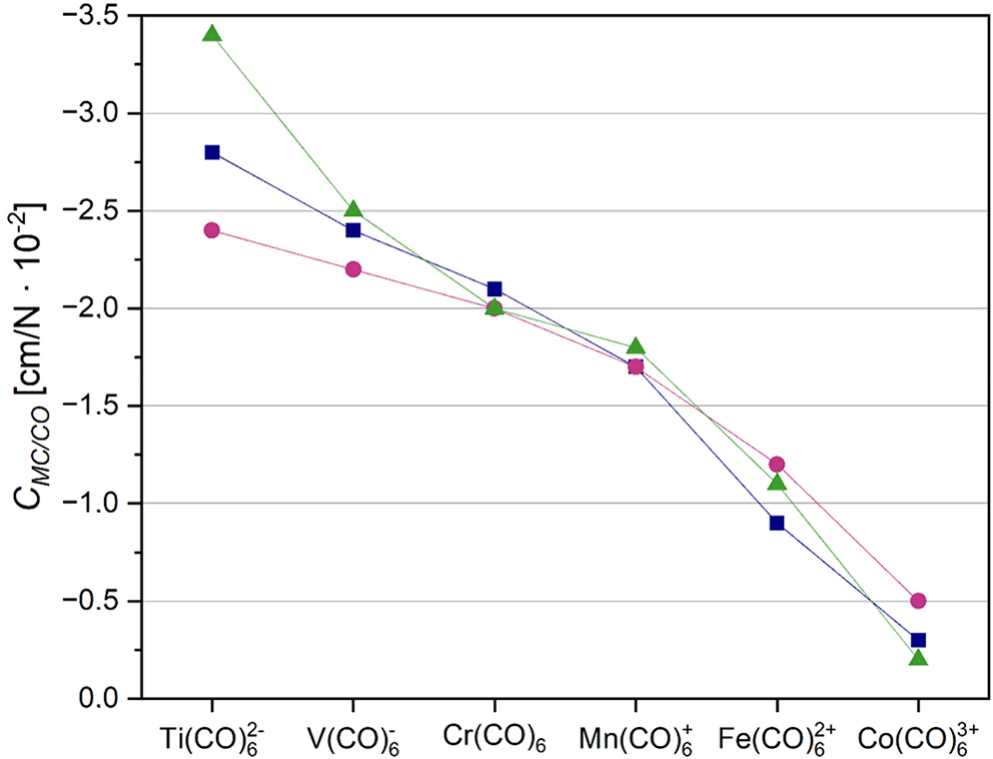
DFT coupling compliance constants in [cm/N × 10^–2^] between adjacent M-C and C–O bonds in isoelectronic 3d hexacarbonyl complexes. The basis set used was def2-TZVPD, while the functionals MN15 (dark blue squares), TPSSh (magenta circles) and ωB97X-D (green triangles) correspond to the three sets of data points shown. The coupling constants correctly describe the decrease of backbonding going from the negatively charged carbonyltitanate Ti­(CO)_6_^2–^ to the highly positively charged Co­(CO)_6_^3+^.

**Figure 8:**
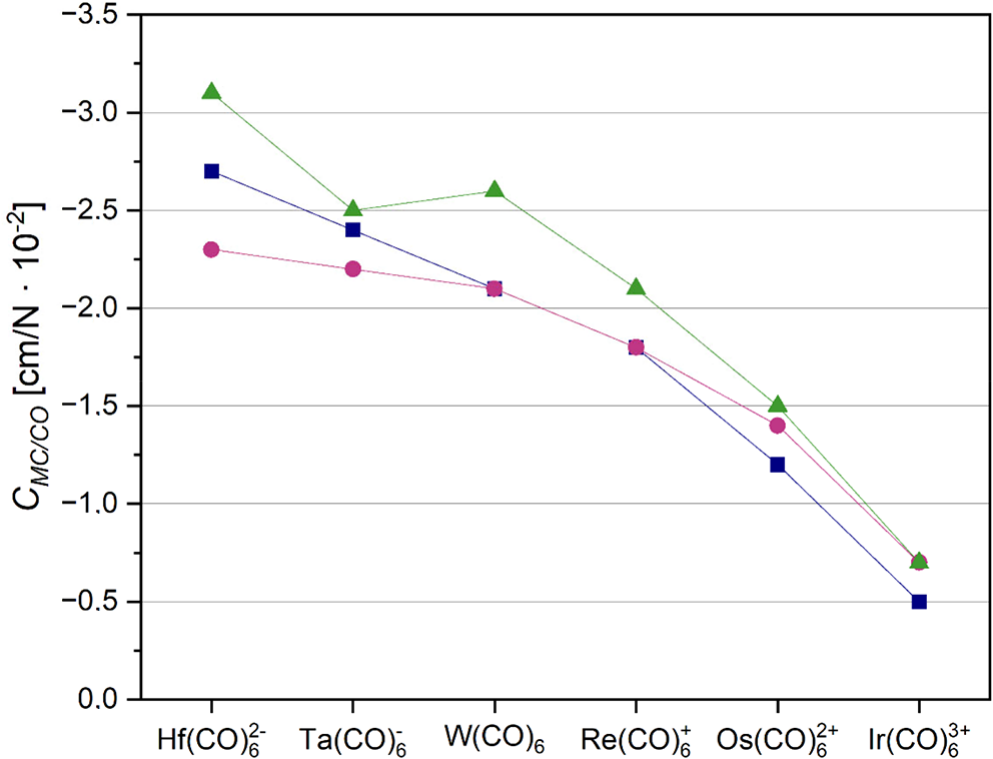
DFT coupling compliance constants in [cm/N × 10^–2^] between adjacent M-C and C–O bonds in isoelectronic 5d hexacarbonyl complexes. The basis set used was def2-TZVPD, while the functionals MN15 (dark blue squares), TPSSh (magenta circles) and ωB97X-D (green triangles) correspond to the three sets of data points shown. The coupling constants correctly describe the decrease of back-bonding going from the negatively charged hafnate Hf­(CO)_6_^2–^ to the highly positively charged Ir­(CO)_6_^3+^.

Using our unique numerical values we are now able to quantify the situation: the synergistic back-bonding is going down to 25% in Ir­(CO)_6_^3+^ (−0.5 cm/N × 10^–2^) compared to the neutral Cr­(CO)_6_ (around −2.0 cm/N × 10^–2^). The same is true for the respective 5d analogues. Again, even if the mean error predicting the compliance coupling constants using the ωB97X-D functional is low, it seems to overemphasize the back-bonding for the Ti­(CO)_6_ dianion, while it underestimates it for the Ta­(CO)_6_ anion at the same time. The description of the electronic situation using TPSSh or the MN15 functional appears to be much more balanced in this special case.

## Conclusions

While force coupling constants could serve as descriptors of electron delocalization in general, the computation of coupling compliance constants might be useful as descriptors of the Dewar–Chatt–Duncanson model in metal carbonyls.

We therefore developed and checked a semiautomated protocol enabling the efficient computation of unique potential coupling constants. In our algorithm, the most time-consuming step is the Moore-Penrose inversion of the Cartesian Hessian matrix. Additionally, our protocol allows for the direct computation of distinct elements of the compliance matrix **C**, which then allows the successive construction of the compliance matrix step by step. Most importantly, the unique prediction of all coupling constants is included in our algorithm.

Several modern density functional (DFT) methods were tested against coupled cluster theory (CCSD­(T)) in order to evaluate their quality in producing reliable matrix *off-diagonal* elements.

Describing the metal–carbon bonds in metal carbonyls, the overall error of most density functionals is below 20% and for the C–O stiffness even below 15%. Concerning the efficacy of density functionals predicting *coupling force constants* the situation is more complex. Using BP86 (in our study the only representative for a functional without HF exchange), the coupling is overemphasized dramatically. This seems to be the result of an overestimation of π-back-bonding using first generation functionals like BP86.

All in all we advocate the use of modern functionals like MN15, the long-range corrected hybrid density functional ωB97X-D or nonempirical functionals like TPSSh when it comes to the computation of accurate potential *coupling constants* in carbonyl complexes in general. A detailed analysis of the relevant MC/MC_trans_ compliance coupling constants as descriptors for the kinetic trans effect in metal carbonyls is in progress.

We implemented a robust, semiautomated algorithm including the computation of all compliance coupling constants as inverse covariant second derivatives, as part of our latest open source COMPLIANCE code.

## Supplementary Material



## Notes

Computational details, data used for the statistical analysis and raw compliance matrix data (PDF)
