# Supplementary material for: Effective Computation of Coupling Force Constants: Metal Carbonyls as a Test Case
Source: J Chem Theory Comput. 2025 Sep 11;21(18):8729–36. doi: 10.1021/acs.jctc.5c00645 (PMC12461792; doi:10.1021/acs.jctc.5c00645)
Supplement: Supplementary file 1 [file ct5c00645_si_001.pdf]

Supporting Information

Effective Computation of Coupling Force Constants: Metal Carbonyls as a Test Case

*Henrik Borgman, Somi Vasisth, Jörg Grunenberg**

Institute of Organic Chemistry, TU Braunschweig, Hagenring 30, 38106 Braunschweig, Germany.

Table of Contents

I. Computational Details

II. Compliance matrices for monocarbonyl complexes

III. Data used for statistical analysis

IV. Compliance matrices and cartesian coordinates for hexacarbonyl complexes

V. References

I. Computational Details

All calculations were computed with the program Gaussian 16,[S1] together with the corresponding graphical interface GaussView 6.[S2] All methods and basis sets were used as included in Gaussian 16, with the exception of the basis set def2-TZVPD,[S3-S5] which was obtained from the website „Basis Set Exchange“[S6-S8]. Compliance Matrices were computed with the program COMPLIANCE.[S9,S10]

For the structures optimised with the following methods, imaginary frequencies were obtained:

$Ti(CO)_6^{2-}$ ωB97X-D (-22 to -24 cm-1)

$Hf(CO)_6^{2-}$ ωB97X-D (-29 to -30 cm-1)

$Hf(CO)_6^{2-}$ MN15 (-15 to -16 cm-1)

II. Compliance matrices for monocarbonyl complexes

The following compliance matrices serve as the data input for the statistical analysis in chapter III with the diagonal elements being inverted. All compliance constants are given in cm/N, while the relaxed force constants are in N/cm.

Table S1. Compliance matrices for closed-shell monocarbonyl complexes (from left to right and top to bottom) VCO^-, CrCO, $MnCO^+$, $FeCO^{2+}$, NiCO, $CuCO^+$, optimised with CCSD(T)/aug-cc-pvtz

	STRE(C,V)	STRE(C,O)
STRE(C,V)	0.322	
STRE(C,O)	-0.026	0.085

	STRE(C,Cr)	STRE(C,O)
STRE(C,Cr)	0.324	
STRE(C,O)	-0.022	0.067

	STRE(C,Mn)	STRE(C,O)
STRE(C,Mn)	0.480	
STRE(C,O)	-0.022	0.056

	STRE(C,Fe)	STRE(C,O)
STRE(C,Fe)	0.622	
STRE(C,O)	-0.001	0.048

	STRE(C,Ni)	STRE(C,O)
STRE(C,Ni)	0.237	
STRE(C,O)	-0.010	0.064

	STRE(C,Cu)	STRE(C,O)
STRE(C,Cu)	0.806	
STRE(C,O)	-0.002	0.050

Table S2. Compliance matrices for closed-shell monocarbonyl complexes (from left to right and top to bottom) VCO^-, CrCO, $MnCO^+$, $FeCO^{2+}$, NiCO, $CuCO^+$, optimised with B2PLYPD3/def2-TZVPD.

	STRE(C,V)	STRE(C,O)
STRE(C,V)	0.270	
STRE(C,O)	-0.030	0.095

	STRE(C,Cr)	STRE(C,O)
STRE(C,Cr)	0.204	
STRE(C,O)	-0.015	0.072

	STRE(C,Mn)	STRE(C,O)
STRE(C,Mn)	0.471	
STRE(C,O)	-0.022	0.056

	STRE(C,Fe)	STRE(C,O)
STRE(C,Fe)	0.603	
STRE(C,O)	-0.002	0.048

	STRE(C,Ni)	STRE(C,O)
STRE(C,Ni)	0.227	
STRE(C,O)	-0.008	0.065

	STRE(C,Cu)	STRE(C,O)
STRE(C,Cu)	0.677	
STRE(C,O)	-0.006	0.050

Table S3. Compliance matrices for closed-shell monocarbonyl complexes (from left to right and top to bottom) VCO^-, CrCO, $MnCO^+$, $FeCO^{2+}$, NiCO, $CuCO^+$, optimised with B3LYP-D3/def2-TZVPD.

	STRE(C,V)	STRE(C,O)
STRE(C,V)	0.309	
STRE(C,O)	-0.030	0.081

	STRE(C,Cr)	STRE(C,O)
STRE(C,Cr)	0.305	
STRE(C,O)	-0.024	0.071

	STRE(C,Mn)	STRE(C,O)
STRE(C,Mn)	0.469	
STRE(C,O)	-0.017	0.052

	STRE(C,Fe)	STRE(C,O)
STRE(C,Fe)	0.605	
STRE(C,O)	-0.003	0.045

	STRE(C,Ni)	STRE(C,O)
STRE(C,Ni)	0.256	
STRE(C,O)	-0.010	0.059

	STRE(C,Cu)	STRE(C,O)
STRE(C,Cu)	0.701	
STRE(C,O)	-0.005	0.047

Table S4. Compliance matrices for closed-shell monocarbonyl complexes (from left to right and top to bottom) VCO^-, CrCO, $MnCO^+$, $FeCO^{2+}$, NiCO, $CuCO^+$, optimised with BP86-D3/def2-TZVPD.

	STRE(C,V)	STRE(C,O)
STRE(C,V)	0.272	
STRE(C,O)	-0.026	0.091

	STRE(C,Cr)	STRE(C,O)
STRE(C,Cr)	0.281	
STRE(C,O)	-0.021	0.078

	STRE(C,Mn)	STRE(C,O)
STRE(C,Mn)	0.349	
STRE(C,O)	-0.018	0.060

	STRE(C,Fe)	STRE(C,O)
STRE(C,Fe)	0.562	
STRE(C,O)	-0.010	0.051

	STRE(C,Ni)	STRE(C,O)
STRE(C,Ni)	0.236	
STRE(C,O)	-0.008	0.065

	STRE(C,Cu)	STRE(C,O)
STRE(C,Cu)	0.521	
STRE(C,O)	-0.006	0.052

Table S5. Compliance matrices for closed-shell monocarbonyl complexes (from left to right and top to bottom) VCO^-, CrCO, $MnCO^+$, $FeCO^{2+}$, NiCO, $CuCO^+$, optimised with MN15/def2-TZVPD.

	STRE(C,V)	STRE(C,O)
STRE(C,V)	0.302	
STRE(C,O)	-0.030	0.077

	STRE(C,Cr)	STRE(C,O)
STRE(C,Cr)	0.299	
STRE(C,O)	-0.020	0.060

	STRE(C,Mn)	STRE(C,O)
STRE(C,Mn)	0.500	
STRE(C,O)	-0.017	0.050

	STRE(C,Fe)	STRE(C,O)
STRE(C,Fe)	0.572	
STRE(C,O)	-0.002	0.044

	STRE(C,Ni)	STRE(C,O)
STRE(C,Ni)	0.265	
STRE(C,O)	-0.010	0.056

	STRE(C,Cu)	STRE(C,O)
STRE(C,Cu)	0.746	
STRE(C,O)	-0.003	0.046

Table S6. Compliance matrices for closed-shell monocarbonyl complexes (from left to right and top to bottom) VCO^-, CrCO, $MnCO^+$, $FeCO^{2+}$, NiCO, $CuCO^+$, optimised with TPSSh/def2-TZVPD.

	STRE(C,V)	STRE(C,O)
STRE(C,V)	0.292	
STRE(C,O)	-0.028	0.083

	STRE(C,Cr)	STRE(C,O)
STRE(C,Cr)	0.285	
STRE(C,O)	-0.022	0.073

	STRE(C,Mn)	STRE(C,O)
STRE(C,Mn)	0.399	
STRE(C,O)	-0.017	0.054

	STRE(C,Fe)	STRE(C,O)
STRE(C,Fe)	0.544	
STRE(C,O)	-0.000	0.053

	STRE(C,Ni)	STRE(C,O)
STRE(C,Ni)	0.237	
STRE(C,O)	-0.009	0.062

	STRE(C,Cu)	STRE(C,O)
STRE(C,Cu)	0.598	
STRE(C,O)	-0.006	0.049

Table S7. Compliance matrices for closed-shell monocarbonyl complexes (from left to right and top to bottom) VCO^-, CrCO, $MnCO^+$, $FeCO^{2+}$, NiCO, $CuCO^+$, optimised with ωB97X-D/def2-TZVPD.

	STRE(C,V)	STRE(C,O)
STRE(C,V)	0.301	
STRE(C,O)	-0.029	0.076

	STRE(C,Cr)	STRE(C,O)
STRE(C,Cr)	0.242	
STRE(C,O)	-0.018	0.067

	STRE(C,Mn)	STRE(C,O)
STRE(C,Mn)	0.394	
STRE(C,O)	-0.013	0.049

	STRE(C,Fe)	STRE(C,O)
STRE(C,Fe)	0.521	
STRE(C,O)	-0.001	0.043

	STRE(C,Ni)	STRE(C,O)
STRE(C,Ni)	0.244	
STRE(C,O)	-0.010	0.056

	STRE(C,Cu)	STRE(C,O)
STRE(C,Cu)	0.634	
STRE(C,O)	-0.004	0.045

Table S8. Compliance matrix for the open-shell monocarbonyl complex $FeCO^+$, optimised with various functional/basis set combinations, as given in the top-left cells of the seven subtables.

CCSD(T)/aug-cc-pvtz	STRE(C,Fe)	STRE(C,O)
STRE(C,Fe)	0.530	
STRE(C,O)	-0.021	0.054

B2PLYPD3/ def2-TZVPD	STRE(C,Fe)	STRE(C,O)
STRE(C,Fe)	0.497	
STRE(C,O)	-0.020	0.054

B3LYP-D3/ def2-TZVPD	STRE(C,Fe)	STRE(C,O)
STRE(C,Fe)	0.481	
STRE(C,O)	-0.016	0.050

BP86-D3/ def2-TZVPD	STRE(C,Fe)	STRE(C,O)
STRE(C,Fe)	0.394	
STRE(C,O)	-0.012	0.056

MN15/ def2-TZVPD	STRE(C,Fe)	STRE(C,O)
STRE(C,Fe)	0.512	
STRE(C,O)	-0.014	0.048

TPSSh/ def2-TZVPD	STRE(C,Fe)	STRE(C,O)
STRE(C,Fe)	0.406	
STRE(C,O)	-0.016	0.053

ωB97X-D/ def2-TZVPD	STRE(C,Fe)	STRE(C,O)
STRE(C,Fe)	0.573	
STRE(C,O)	-0.012	0.048

III. Data used for statistical analysis

Table S9. Relaxed Force Constants F_{MC} of monocarbonyl complexes as inverted Compliance Constants.

	VCO^-	CrCO	$MnCO^+$	$FeCO^{2+}$	NiCO	$CuCO^+$	$FeCO^+$
CCSD(T)	3.106	3.086	2.083	1.608	4.219	1.241	1.887
B2PLYPD3	3.704	4.902	2.123	1.658	4.405	1.477	2.012
B3LYP-D3	3.236	3.279	2.132	1.653	3.906	1.427	2.079
BP86-D3	3.676	3.559	2.865	1.779	4.237	1.919	2.538
MN15	3.311	3.344	2.000	1.748	3.774	1.340	1.953
TPSSh	3.425	3.509	2.506	1.838	4.219	1.672	2.463
ωB97X-D	3.322	4.132	2.538	1.919	4.098	1.577	1.745

Table S10. Relative error of F_{MC} value from DFT methods with CCSD(T) reference. (font size reduced to ensure table fits on page).

	VCO^-	CrCO	$MnCO^+$	$FeCO^{2+}$	NiCO	$CuCO^+$	$FeCO^+$	Mean Signed Error	Mean Absolute Error
B2PLYPD3	19.25%	58.85%	1.92%	3.11%	4.41%	19.02%	6.62%	16.17%	16.17%
B3LYP-D3	4.19%	6.25%	2.35%	2.80%	-7.42%	14.99%	10.17%	4.76%	6.88%
BP86-D3	18.35%	15.33%	37.54%	10.63%	0.43%	54.63%	34.50%	24.49%	24.49%
MN15	6.60%	8.36%	-3.98%	8.71%	-10.55%	7.98%	3.50%	2.94%	7.10%
TPSSh	10.27%	13.71%	20.31%	14.30%	0.00%	34.73%	30.52%	17.69%	17.69%
ωB97X-D	6.95%	33.90%	21.84%	19.34%	-2.87%	27.07%	-7.53%	14.10%	17.07%

Table S11. Relaxed Force Constants F_{CO} of monocarbonyl complexes as inverted Compliance Constants.

	VCO^-	CrCO	$MnCO^+$	$FeCO^{2+}$	NiCO	$CuCO^+$	$FeCO^+$
CCSD(T)	11.765	14.925	17.857	20.833	15.625	20.000	18.519
B2PLYPD3	10.526	13.889	17.857	20.833	15.385	20.000	18.519
B3LYP-D3	12.346	14.085	19.231	23.872	16.949	21.277	20.000
BP86-D3	10.989	12.821	16.667	19.608	15.385	19.231	17.857
MN15	12.987	16.667	20.000	22.727	17.857	21.739	20.833
TPSSh	12.048	13.699	18.519	18.868	16.129	20.408	18.868
ωB97X-D	13.158	14.925	20.408	23.256	17.857	22.222	20.833

Table S12. Relative error of F_{CO} value from DFT methods with CCSD(T) reference. (font size reduced to ensure table fits on page).

	VCO^-	CrCO	$MnCO^+$	$FeCO^{2+}$	NiCO	$CuCO^+$	$FeCO^+$	Mean Signed Error	Mean Absolute Error
B2PLYPD3	-10.53%	-6.94%	0.00%	0.00%	-1.54%	0.00%	0.00%	-2.72%	2.72%
B3LYP-D3	4.94%	-5.63%	7.69%	14.59%	8.47%	6.39%	8.00%	6.35%	7.96%
BP86-D3	-6.60%	-14.10%	-6.66%	-5.88%	-1.54%	-3.84%	-3.57%	-6.03%	6.03%
MN15	10.39%	11.67%	12.00%	9.09%	14.28%	8.70%	12.50%	11.23%	11.23%
TPSSh	2.41%	-8.21%	3.71%	-9.43%	3.23%	2.04%	1.88%	-0.63%	4.42%
ωB97X-D	11.84%	0.00%	14.29%	11.63%	14.28%	11.11%	12.50%	10.81%	10.81%

Table S13. Compliance coupling constants $C_{MC/CO}$ of monocarbonyl complexes.

	VCO^-	CrCO	$MnCO^+$	$FeCO^{2+}$	NiCO	$CuCO^+$	$FeCO^+$
CCSD(T)	-0.026	-0.022	-0.022	-0.001	-0.010	-0.002	-0.021
B2PLYPD3	-0.030	-0.015	-0.022	-0.002	-0.008	-0.006	-0.020
B3LYP-D3	-0.030	-0.024	-0.017	-0.003	-0.010	-0.005	-0.016
BP86-D3	-0.026	-0.021	-0.018	-0.01	-0.008	-0.006	-0.012
MN15	-0.030	-0.020	-0.017	-0.002	-0.010	-0.003	-0.014
TPSSh	-0.028	-0.022	-0.017	0.000	-0.009	-0.006	-0.016
ωB97X-D	-0.029	-0.018	-0.013	-0.001	-0.010	-0.004	-0.012

Table S14. Relative error of $C_{MC/CO}$ value from DFT methods with CCSD(T) reference (font size reduced to ensure table fits on page).

	VCO^-	CrCO	$MnCO^+$	$FeCO^{2+}$	NiCO	$CuCO^+$	$FeCO^+$	Mean Signed Error	Mean Absolute Error
B2PLYPD3	15.38%	-31.82%	0.00%	100.00%	-20.00%	200.00%	-4.76%	36.97%	53.14%
B3LYP-D3	15.38%	9.09%	-22.73%	200.00%	0.00%	150.00%	-23.81%	46.85%	60.14%
BP86-D3	0.00%	-4.55%	-18.18%	900.00%	-20.00%	200.00%	-42.86%	144.92%	169.37%
MN15	15.38%	-9.09%	-22.73%	100.00%	0.00%	50.00%	-33.33%	14.32%	32.93%
TPSSh	7.69%	0.00%	-22.73%	-100.00%	-10.00%	200.00%	-23.81%	7.31%	52.03%
ωB97X-D	11.54%	-18.18%	-40.91%	0.00%	0.00%	100.00%	-42.86%	1.37%	30.50%

IV. Compliance matrices and cartesian coordinates for the DFT-optimised hexacarbonyl complexes

Table S15. Cartesian Coordinates and compliance matrix for $Ti(CO)_6^{2-}$ (TPSSh/def2-TZVPD)

C	0.007112	0.019436	2.034134
O	0.007375	0.021027	3.212767
C	0.006999	2.072355	-0.018813
O	0.007752	3.251017	-0.018678
C	0.007096	0.023080	-2.073396
O	0.007184	0.026358	-3.252042
C	2.060149	0.020630	-0.021862
O	3.238794	0.022984	-0.023372
C	-2.047366	0.020648	-0.021456
O	-3.226002	0.022646	-0.023447
C	0.006909	-2.035188	-0.021366
O	0.007528	-3.213809	-0.022673
Ti	0.006470	0.018816	-0.019797

	STRE(C,O)	STRE(C,Ti)
STRE(C,O)	0.076	
STRE(C,Ti)	-0.024	0.541

Table S16. Cartesian Coordinates and compliance matrix for $Ti(CO)_6^{2-}$ (MN15/def2-TZVPD)

C	0.007222	0.019604	2.027628
O	0.007133	0.021014	3.201667
C	0.007230	2.066359	-0.019135
O	0.007647	3.240414	-0.019016
C	0.006893	0.022762	-2.067511
O	0.007209	0.026616	-3.241547
C	2.054163	0.020645	-0.021678
O	3.228200	0.023019	-0.023884
C	-2.040992	0.020549	-0.021408
O	-3.215029	0.021899	-0.022324
C	0.006778	-2.028881	-0.021041
O	0.006913	-3.202921	-0.021738
Ti	0.006631	0.018922	-0.020013

	STRE(C,O)	STRE(C,Ti)
STRE(C,O)	0.071	
STRE(C,Ti)	-0.028	0.558

Table S17. Cartesian Coordinates and compliance matrix for $Ti(CO)_6^{2-}$ (ωB97X-D/def2-TZVPD)

C	0.007262	0.020394	2.026515
O	0.006778	0.023470	3.195388
C	0.007031	2.063844	-0.019275
O	0.007795	3.232772	-0.019407
C	0.006885	0.022603	-2.065585
O	0.007678	0.026243	-3.234479
C	2.052395	0.020575	-0.021907
O	3.221285	0.023755	-0.024440
C	-2.039667	0.020581	-0.021498
O	-3.208545	0.022888	-0.023619
C	0.006959	-2.028236	-0.020636
O	0.007690	-3.197094	-0.021345
Ti	0.006452	0.018206	-0.019711

	STRE(C,O)	STRE(C,Ti)
STRE(C,O)	0.071	
STRE(C,Ti)	-0.034	0.603

Table S18. Cartesian Coordinates and compliance matrix for $V(CO)_6^{-}$ (TPSSh/def2-TZVPD)

C	0.000000	0.000000	1.960031
O	0.000000	0.000000	3.120404
C	-0.000000	-1.960031	-0.000000
O	-0.000000	-3.120404	-0.000000
C	-0.000000	0.000000	-1.960031
O	-0.000000	0.000000	-3.120404
C	-1.960031	0.000000	0.000000
O	-3.120404	0.000000	0.000000
C	1.960031	-0.000000	-0.000000
O	3.120404	-0.000000	-0.000000
C	0.000000	1.960031	-0.000000
O	0.000000	3.120404	-0.000000
V	-0.000000	0.000000	-0.000000

	STRE(C,O)	STRE(C,V)
STRE(C,O)	0.066	
STRE(C,V)	-0.022	0.445

Table S19. Cartesian Coordinates and compliance matrix for $V(CO)_6^-$ (MN15/def2-TZVPD)

C	0.000000	0.000000	1.954985
O	0.000000	0.000000	3.111130
C	-0.000000	-1.954985	-0.000000
O	-0.000000	-3.111130	-0.000000
C	-0.000000	0.000000	-1.954985
O	-0.000000	0.000000	-3.111130
C	-1.954985	0.000000	0.000000
O	-3.111130	0.000000	0.000000
C	1.954985	-0.000000	-0.000000
O	3.111130	-0.000000	-0.000000
C	0.000000	1.954985	-0.000000
O	0.000000	3.111130	-0.000000
V	-0.000000	0.000000	-0.000000

	STRE(C,O)	STRE(C,V)
STRE(C,O)	0.062	
STRE(C,V)	-0.024	0.465

Table S20. Cartesian Coordinates and compliance matrix for $V(CO)_6^-$ (ωB97X-D/def2-TZVPD)

C	0.000000	0.000000	1.952884
O	0.000000	0.000000	3.104166
C	-0.000000	-1.952884	-0.000000
O	-0.000000	-3.104166	-0.000000
C	-0.000000	0.000000	-1.952884
O	-0.000000	0.000000	-3.104166
C	-1.952884	0.000000	0.000000
O	-3.104166	0.000000	0.000000
C	1.952884	-0.000000	-0.000000
O	3.104166	-0.000000	-0.000000
C	0.000000	1.952884	-0.000000
O	0.000000	3.104166	-0.000000
V	-0.000000	0.000000	-0.000000

	STRE(C,O)	STRE(C,V)
STRE(C,O)	0.062	
STRE(C,V)	-0.025	0.457

Table S21. Cartesian Coordinates and compliance matrix for $Cr(CO)_6$ (TPSSh/def2-TZVPD)

C	0.000000	0.000000	1.911903
O	0.000000	0.000000	3.055673
C	-0.000000	-1.911903	-0.000000
O	-0.000000	-3.055673	-0.000000
C	-0.000000	0.000000	-1.911903
O	-0.000000	0.000000	-3.055673
C	-1.911903	0.000000	0.000000
O	-3.055673	0.000000	0.000000
C	1.911903	-0.000000	-0.000000
O	3.055673	-0.000000	-0.000000
C	0.000000	1.911903	-0.000000
O	0.000000	3.055673	-0.000000
Cr	-0.000000	0.000000	-0.000000

	STRE(C,O)	STRE(C,Cr)
STRE(C,O)	0.059	
STRE(C,Cr)	-0.020	0.446

Table S22. Cartesian Coordinates and compliance matrix for $Cr(CO)_6$ (MN15/def2-TZVPD)

C	0.000000	0.000000	1.910514
O	0.000000	0.000000	3.050090
C	-0.000000	-1.910514	-0.000000
O	-0.000000	-3.050090	-0.000000
C	-0.000000	0.000000	-1.910514
O	-0.000000	0.000000	-3.050090
C	-1.910514	0.000000	0.000000
O	-3.050090	0.000000	0.000000
C	1.910514	-0.000000	-0.000000
O	3.050090	-0.000000	-0.000000
C	0.000000	1.910514	-0.000000
O	0.000000	3.050090	-0.000000
Cr	-0.000000	0.000000	-0.000000

	STRE(C,O)	STRE(C,Cr)
STRE(C,O)	0.055	
STRE(C,Cr)	-0.021	0.476

Table S23. Cartesian Coordinates and compliance matrix for $Cr(CO)_6$ (ωB97X-D/def2-TZVPD)

C	0.000000	0.000000	1.910886
O	0.000000	0.000000	3.045807
C	-0.000000	-1.910886	-0.000000
O	-0.000000	-3.045807	-0.000000
C	-0.000000	0.000000	-1.910886
O	-0.000000	0.000000	-3.045807
C	-1.910886	0.000000	0.000000
O	-3.045807	0.000000	0.000000
C	1.910886	-0.000000	-0.000000
O	3.045807	-0.000000	-0.000000
C	0.000000	1.910886	-0.000000
O	0.000000	3.045807	-0.000000
Cr	-0.000000	-0.000000	-0.000000

	STRE(C,O)	STRE(C,Cr)
STRE(C,O)	0.054	
STRE(C,Cr)	-0.020	0.427

Table S24. Cartesian Coordinates and compliance matrix for $Mn(CO)_6^+$ (TPSSh/def2-TZVPD)

C	0.000000	0.000000	1.899170
O	0.000000	0.000000	3.029027
C	-0.000000	-1.899170	-0.000000
O	-0.000000	-3.029027	-0.000000
C	-0.000000	0.000000	-1.899170
O	-0.000000	0.000000	-3.029027
C	-1.899170	0.000000	0.000000
O	-3.029027	0.000000	0.000000
C	1.899170	-0.000000	-0.000000
O	3.029027	-0.000000	-0.000000
C	0.000000	1.899170	-0.000000
O	0.000000	3.029027	-0.000000
Mn	-0.000000	0.000000	-0.000000

	STRE(C,O)	STRE(C,Mn)
STRE(C,O)	0.053	
STRE(C,Mn)	-0.017	0.506

Table S25. Cartesian Coordinates and compliance matrix for $Mn(CO)_6^+$ (MN15/def2-TZVPD)

C	0.000000	0.000000	1.904979
O	0.000000	0.000000	3.030709
C	-0.000000	-1.904979	-0.000000
O	-0.000000	-3.030709	-0.000000
C	-0.000000	0.000000	-1.904979
O	-0.000000	0.000000	-3.030709
C	-1.904979	0.000000	0.000000
O	-3.030709	0.000000	0.000000
C	1.904979	-0.000000	-0.000000
O	3.030709	-0.000000	-0.000000
C	0.000000	1.904979	-0.000000
O	0.000000	3.030709	-0.000000
Mn	-0.000000	0.000000	-0.000000

	STRE(C,O)	STRE(C,Mn)
STRE(C,O)	0.049	
STRE(C,Mn)	-0.017	0.551

Table S26. Cartesian Coordinates and compliance matrix for $Mn(CO)_6^+$ (ωB97X-D/def2-TZVPD)

C	0.000000	0.000000	1.908935
O	0.000000	0.000000	3.030074
C	-0.000000	-1.908935	-0.000000
O	-0.000000	-3.030074	-0.000000
C	-0.000000	0.000000	-1.908935
O	-0.000000	0.000000	-3.030074
C	-1.908935	0.000000	0.000000
O	-3.030074	0.000000	0.000000
C	1.908935	-0.000000	-0.000000
O	3.030074	-0.000000	-0.000000
C	0.000000	1.908935	-0.000000
O	0.000000	3.030074	-0.000000
Mn	-0.000000	-0.000000	-0.000000

	STRE(C,O)	STRE(C,Mn)
STRE(C,O)	0.049	
STRE(C,Mn)	-0.018	0.561

Table S27. Cartesian Coordinates and compliance matrix for $Fe(CO)_6^{2+}$ (TPSSh/def2-TZVPD)

C	0.000000	0.000000	1.916398
O	0.000000	0.000000	3.035956
C	-0.000000	-1.916398	-0.000000
O	-0.000000	-3.035956	-0.000000
C	-0.000000	0.000000	-1.916398
O	-0.000000	0.000000	-3.035956
C	-1.916398	0.000000	0.000000
O	-3.035956	0.000000	0.000000
C	1.916398	-0.000000	-0.000000
O	3.035956	-0.000000	-0.000000
C	0.000000	1.916398	-0.000000
O	0.000000	3.035956	-0.000000
Fe	0.000000	0.000000	-0.000000

	STRE(C,O)	STRE(C,Fe)
STRE(C,O)	0.049	
STRE(C,Fe)	-0.012	0.611

Table S28. Cartesian Coordinates and compliance matrix for $Fe(CO)_6^{2+}$ (MN15/def2-TZVPD)

C	0.000000	0.000000	1.923528
O	0.000000	0.000000	3.039163
C	-0.000000	-1.923528	-0.000000
O	-0.000000	-3.039163	-0.000000
C	-0.000000	0.000000	-1.923528
O	-0.000000	0.000000	-3.039163
C	-1.923528	0.000000	0.000000
O	-3.039163	0.000000	0.000000
C	1.923528	-0.000000	-0.000000
O	3.039163	-0.000000	-0.000000
C	0.000000	1.923528	-0.000000
O	0.000000	3.039163	-0.000000
Fe	0.000000	-0.000000	-0.000000

	STRE(C,O)	STRE(C,Fe)
STRE(C,O)	0.046	
STRE(C,Fe)	-0.009	0.637

Table S29. Cartesian Coordinates and compliance matrix for $Fe(CO)_6^{2+}$ (ωB97X-D/def2-TZVPD)

C	0.000000	0.000000	1.946469
O	0.000000	0.000000	3.057286
C	-0.000000	-1.946469	-0.000000
O	-0.000000	-3.057286	-0.000000
C	-0.000000	0.000000	-1.946469
O	-0.000000	0.000000	-3.057286
C	-1.946469	0.000000	0.000000
O	-3.057286	0.000000	0.000000
C	1.946469	-0.000000	-0.000000
O	3.057286	-0.000000	-0.000000
C	0.000000	1.946469	-0.000000
O	0.000000	3.057286	-0.000000
Fe	-0.000000	0.000000	-0.000000

	STRE(C,O)	STRE(C,Fe)
STRE(C,O)	0.045	
STRE(C,Fe)	-0.011	0.847

Table S30. Cartesian Coordinates and compliance matrix for $Co(CO)_6^{3+}$ (TPSSh/def2-TZVPD)

C	0.000000	0.000000	1.947841
O	0.000000	0.000000	3.061622
C	-0.000000	-1.947841	-0.000000
O	-0.000000	-3.061622	-0.000000
C	-0.000000	0.000000	-1.947841
O	-0.000000	0.000000	-3.061622
C	-1.947841	0.000000	0.000000
O	-3.061622	0.000000	0.000000
C	1.947841	-0.000000	-0.000000
O	3.061622	-0.000000	-0.000000
C	0.000000	1.947841	-0.000000
O	0.000000	3.061622	-0.000000
Co	0.000000	0.000000	-0.000000

	STRE(C,O)	STRE(C,Co)
STRE(C,O)	0.047	
STRE(C,Co)	-0.005	0.675

Table S31. Cartesian Coordinates and compliance matrix for $Co(CO)_6^{3+}$ (MN15/def2-TZVPD)

C	0.000000	0.000000	1.940496
O	0.000000	0.000000	3.050366
C	-0.000000	-1.940496	-0.000000
O	-0.000000	-3.050366	-0.000000
C	-0.000000	0.000000	-1.940496
O	-0.000000	0.000000	-3.050366
C	-1.940496	0.000000	0.000000
O	-3.050366	0.000000	0.000000
C	1.940496	-0.000000	-0.000000
O	3.050366	-0.000000	-0.000000
C	0.000000	1.940496	-0.000000
O	0.000000	3.050366	-0.000000
Co	0.000000	-0.000000	-0.000000

	STRE(C,O)	STRE(C,Co)
STRE(C,O)	0.044	
STRE(C,Co)	-0.003	0.628

Table S32. Cartesian Coordinates and compliance matrix for $Co(CO)_6^{3+}$ (ωB97X-D/def2-TZVPD)

C	0.000000	0.000000	1.965663
O	0.000000	0.000000	3.070995
C	-0.000000	-1.965663	-0.000000
O	-0.000000	-3.070995	-0.000000
C	-0.000000	0.000000	-1.965663
O	-0.000000	0.000000	-3.070995
C	-1.965663	0.000000	0.000000
O	-3.070995	0.000000	0.000000
C	1.965663	-0.000000	-0.000000
O	3.070995	-0.000000	-0.000000
C	0.000000	1.965663	-0.000000
O	0.000000	3.070995	-0.000000
Co	0.000000	-0.000000	-0.000000

	STRE(C,O)	STRE(C,Co)
STRE(C,O)	0.044	
STRE(C,Co)	-0.002	0.665

Table S33. Cartesian Coordinates and compliance matrix for $Hf(CO)_6^{2-}$ (TPSSh/def2-TZVPD)

C	0.000000	0.000000	2.222510
O	0.000000	0.000000	3.400327
C	-0.000000	-2.222510	-0.000000
O	-0.000000	-3.400327	-0.000000
C	-0.000000	0.000000	-2.222510
O	-0.000000	0.000000	-3.400327
C	-2.222510	0.000000	0.000000
O	-3.400327	0.000000	0.000000
C	2.222510	-0.000000	-0.000000
O	3.400327	-0.000000	-0.000000
C	0.000000	2.222510	-0.000000
O	0.000000	3.400327	-0.000000
Hf	-0.000000	0.000000	-0.000000

	STRE(C,O)	STRE(C,Hf)
STRE(C,O)	0.075	
STRE(C,Hf)	-0.023	0.584

Table S34. Cartesian Coordinates and compliance matrix for $Hf(CO)_6^{2-}$ (MN15/def2-TZVPD)

C	0.000000	0.000000	2.221191
O	0.000000	0.000000	3.393652
C	-0.000000	-2.221191	-0.000000
O	-0.000000	-3.393652	-0.000000
C	-0.000000	0.000000	-2.221191
O	-0.000000	0.000000	-3.393652
C	-2.221191	0.000000	0.000000
O	-3.393652	0.000000	0.000000
C	2.221191	-0.000000	-0.000000
O	3.393652	-0.000000	-0.000000
C	0.000000	2.221191	-0.000000
O	0.000000	3.393652	-0.000000
Hf	-0.000000	0.000000	-0.000000

	STRE(C,O)	STRE(C,Hf)
STRE(C,O)	0.071	
STRE(C,Hf)	-0.027	0.615

Table S35. Cartesian Coordinates and compliance matrix for $Hf(CO)_6^{2-}$ (ωB97X-D/def2-TZVPD)

C	0.000000	0.000000	2.217018
O	0.000000	0.000000	3.384568
C	-0.000000	-2.217018	-0.000000
O	-0.000000	-3.384568	-0.000000
C	-0.000000	0.000000	-2.217018
O	-0.000000	0.000000	-3.384568
C	-2.217018	0.000000	0.000000
O	-3.384568	0.000000	0.000000
C	2.217018	-0.000000	-0.000000
O	3.384568	-0.000000	-0.000000
C	0.000000	2.217018	-0.000000
O	0.000000	3.384568	-0.000000
Hf	-0.000000	-0.000000	-0.000000

	STRE(C,O)	STRE(C,Hf)
STRE(C,O)	0.071	
STRE(C,Hf)	-0.031	0.628

Table S36. Cartesian Coordinates and compliance matrix for $Ta(CO)_6^{-}$ (TPSSh/def2-TZVPD)

C	0.000000	0.000000	2.127954
O	0.000000	0.000000	3.288443
C	-0.000000	-2.127954	-0.000000
O	-0.000000	-3.288443	-0.000000
C	-0.000000	0.000000	-2.127954
O	-0.000000	0.000000	-3.288443
C	-2.127954	0.000000	0.000000
O	-3.288443	0.000000	0.000000
C	2.127954	-0.000000	-0.000000
O	3.288443	-0.000000	-0.000000
C	0.000000	2.127954	-0.000000
O	0.000000	3.288443	-0.000000
Ta	-0.000000	-0.000000	-0.000000

	STRE(C,O)	STRE(C,Ta)
STRE(C,O)	0.066	
STRE(C,Hf)	-0.022	0.465

Table S37. Cartesian Coordinates and compliance matrix for $Ta(CO)_6^-$ (MN15/def2-TZVPD)

C	0.000000	0.000000	2.126922
O	0.000000	0.000000	3.282829
C	-0.000000	-2.126922	-0.000000
O	-0.000000	-3.282829	-0.000000
C	-0.000000	0.000000	-2.126922
O	-0.000000	0.000000	-3.282829
C	-2.126922	0.000000	0.000000
O	-3.282829	0.000000	0.000000
C	2.126922	-0.000000	-0.000000
O	3.282829	-0.000000	-0.000000
C	0.000000	2.126922	-0.000000
O	0.000000	3.282829	-0.000000
Ta	-0.000000	0.000000	-0.000000

	STRE(C,O)	STRE(C,Ta)
STRE(C,O)	0.062	
STRE(C,Hf)	-0.024	0.487

Table S38. Cartesian Coordinates and compliance matrix for $Ta(CO)_6^-$ (ωB97X-D/def2-TZVPD)

C	0.000000	0.000000	2.125983
O	0.000000	0.000000	3.276971
C	-0.000000	-2.125983	-0.000000
O	-0.000000	-3.276971	-0.000000
C	-0.000000	0.000000	-2.125983
O	-0.000000	0.000000	-3.276971
C	-2.125983	0.000000	0.000000
O	-3.276971	0.000000	0.000000
C	2.125983	-0.000000	-0.000000
O	3.276971	-0.000000	-0.000000
C	0.000000	2.125983	-0.000000
O	0.000000	3.276971	-0.000000
Ta	-0.000000	0.000000	-0.000000

	STRE(C,O)	STRE(C,Ta)
STRE(C,O)	0.062	
STRE(C,Hf)	-0.025	0.469

Table S39. Cartesian Coordinates and compliance matrix for $W(CO)_6$ (TPSSh/def2-TZVPD)

C	0.000000	0.000000	2.072160
O	0.000000	0.000000	3.217010
C	-0.000000	-2.072160	-0.000000
O	-0.000000	-3.217010	-0.000000
C	-0.000000	0.000000	-2.072160
O	-0.000000	0.000000	-3.217010
C	-2.072160	0.000000	0.000000
O	-3.217010	0.000000	0.000000
C	2.072160	-0.000000	-0.000000
O	3.217010	-0.000000	-0.000000
C	0.000000	2.072160	-0.000000
O	0.000000	3.217010	-0.000000
W	0.000000	0.000000	-0.000000

	STRE(C,O)	STRE(C,W)
STRE(C,O)	0.059	
STRE(C,W)	-0.021	0.441

Table S40. Cartesian Coordinates and compliance matrix for $W(CO)_6$ (MN15/def2-TZVPD)

C	0.000000	0.000000	2.072262
O	0.000000	0.000000	3.212936
C	-0.000000	-2.072262	-0.000000
O	-0.000000	-3.212936	-0.000000
C	-0.000000	0.000000	-2.072262
O	-0.000000	0.000000	-3.212936
C	-2.072262	0.000000	0.000000
O	-3.212936	0.000000	0.000000
C	2.072262	-0.000000	-0.000000
O	3.212936	-0.000000	-0.000000
C	0.000000	2.072262	-0.000000
O	0.000000	3.212936	-0.000000
W	0.000000	0.000000	-0.000000

	STRE(C,O)	STRE(C,W)
STRE(C,O)	0.055	
STRE(C,W)	-0.021	0.463

Table S41. Cartesian Coordinates and compliance matrix for $W(CO)_6$ (ωB97X-D/def2-TZVPD)

C	0.000000	0.000000	2.071593
O	0.000000	0.000000	3.207528
C	-0.000000	-2.071593	-0.000000
O	-0.000000	-3.207528	-0.000000
C	-0.000000	0.000000	-2.071593
O	-0.000000	0.000000	-3.207528
C	-2.071593	0.000000	0.000000
O	-3.207528	0.000000	0.000000
C	2.071593	-0.000000	-0.000000
O	3.207528	-0.000000	-0.000000
C	0.000000	2.071593	-0.000000
O	0.000000	3.207528	-0.000000
W	0.000000	0.000000	-0.000000

	STRE(C,O)	STRE(C,W)
STRE(C,O)	0.055	
STRE(C,W)	-0.026	0.536

Table S42. Cartesian Coordinates and compliance matrix for $Re(CO)_6^+$ (TPSSh/def2-TZVPD)

C	0.000000	0.000000	2.045437
O	0.000000	0.000000	3.176936
C	-0.000000	-2.045437	-0.000000
O	-0.000000	-3.176936	-0.000000
C	-0.000000	0.000000	-2.045437
O	-0.000000	0.000000	-3.176936
C	-2.045437	0.000000	0.000000
O	-3.176936	0.000000	0.000000
C	2.045437	-0.000000	-0.000000
O	3.176936	-0.000000	-0.000000
C	0.000000	2.045437	-0.000000
O	0.000000	3.176936	-0.000000
Re	0.000000	-0.000000	-0.000000

	STRE(C,O)	STRE(C,Re)
STRE(C,O)	0.054	
STRE(C,Re)	-0.018	0.470

Table S43. Cartesian Coordinates and compliance matrix for $Re(CO)_6^+$ (MN15/def2-TZVPD)

C	0.000000	0.000000	2.046726
O	0.000000	0.000000	3.174299
C	-0.000000	-2.046726	-0.000000
O	-0.000000	-3.174299	-0.000000
C	-0.000000	0.000000	-2.046726
O	-0.000000	0.000000	-3.174299
C	-2.046726	0.000000	0.000000
O	-3.174299	0.000000	0.000000
C	2.046726	-0.000000	-0.000000
O	3.174299	-0.000000	-0.000000
C	0.000000	2.046726	-0.000000
O	0.000000	3.174299	-0.000000
Re	0.000000	0.000000	-0.000000

	STRE(C,O)	STRE(C,Re)
STRE(C,O)	0.050	
STRE(C,Re)	-0.018	0.485

Table S44. Cartesian Coordinates and compliance matrix for $Re(CO)_6^+$ (ωB97X-D/def2-TZVPD)

C	0.000000	0.000000	2.044708
O	0.000000	0.000000	3.167642
C	-0.000000	-2.044708	-0.000000
O	-0.000000	-3.167642	-0.000000
C	-0.000000	0.000000	-2.044708
O	-0.000000	0.000000	-3.167642
C	-2.044708	0.000000	0.000000
O	-3.167642	0.000000	0.000000
C	2.044708	-0.000000	-0.000000
O	3.167642	-0.000000	-0.000000
C	0.000000	2.044708	-0.000000
O	0.000000	3.167642	-0.000000
Re	0.000000	0.000000	-0.000000

	STRE(C,O)	STRE(C,Re)
STRE(C,O)	0.050	
STRE(C,Re)	-0.021	0.558

Table S45. Cartesian Coordinates and compliance matrix for $Os(CO)_6^{2+}$ (TPSSh/def2-TZVPD)

C	0.000000	0.000000	2.042978
O	0.000000	0.000000	3.163941
C	-0.000000	-2.042978	-0.000000
O	-0.000000	-3.163941	-0.000000
C	-0.000000	0.000000	-2.042978
O	-0.000000	0.000000	-3.163941
C	-2.042978	0.000000	0.000000
O	-3.163941	0.000000	0.000000
C	2.042978	-0.000000	-0.000000
O	3.163941	-0.000000	-0.000000
C	0.000000	2.042978	-0.000000
O	0.000000	3.163941	-0.000000
Os	0.000000	0.000000	-0.000000

	STRE(C,O)	STRE(C,Os)
STRE(C,O)	0.049	
STRE(C,Os)	-0.014	0.534

Table S46. Cartesian Coordinates and compliance matrix for $Os(CO)_6^{2+}$ (MN15/def2-TZVPD)

C	0.000000	0.000000	2.044251
O	0.000000	0.000000	3.161517
C	-0.000000	-2.044251	-0.000000
O	-0.000000	-3.161517	-0.000000
C	-0.000000	0.000000	-2.044251
O	-0.000000	0.000000	-3.161517
C	-2.044251	0.000000	0.000000
O	-3.161517	0.000000	0.000000
C	2.044251	-0.000000	-0.000000
O	3.161517	-0.000000	-0.000000
C	0.000000	2.044251	-0.000000
O	0.000000	3.161517	-0.000000
Os	0.000000	-0.000000	-0.000000

	STRE(C,O)	STRE(C,Os)
STRE(C,O)	0.046	
STRE(C,Os)	-0.012	0.528

Table S47. Cartesian Coordinates and compliance matrix for $Os(CO)_6^{2+}$ (ωB97X-D/def2-TZVPD)

C	0.000000	0.000000	2.044781
O	0.000000	0.000000	3.157670
C	-0.000000	-2.044781	-0.000000
O	-0.000000	-3.157670	-0.000000
C	-0.000000	0.000000	-2.044781
O	-0.000000	0.000000	-3.157670
C	-2.044781	0.000000	0.000000
O	-3.157670	0.000000	0.000000
C	2.044781	-0.000000	-0.000000
O	3.157670	-0.000000	-0.000000
C	0.000000	2.044781	-0.000000
O	0.000000	3.157670	-0.000000
Os	0.000000	0.000000	-0.000000

	STRE(C,O)	STRE(C,Os)
STRE(C,O)	0.046	
STRE(C,Os)	-0.015	0.631

Table S48. Cartesian Coordinates and compliance matrix for $Ir(CO)_6^{3+}$ (TPSSh/def2-TZVPD)

C	0.000000	0.000000	2.059383
O	0.000000	0.000000	3.173770
C	-0.000000	-2.059383	-0.000000
O	-0.000000	-3.173770	-0.000000
C	-0.000000	0.000000	-2.059383
O	-0.000000	0.000000	-3.173770
C	-2.059383	0.000000	0.000000
O	-3.173770	0.000000	0.000000
C	2.059383	-0.000000	-0.000000
O	3.173770	-0.000000	-0.000000
C	0.000000	2.059383	-0.000000
O	0.000000	3.173770	-0.000000
Ir	0.000000	-0.000000	-0.000000

	STRE(C,O)	STRE(C,Ir)
STRE(C,O)	0.047	
STRE(C,Ir)	-0.007	0.575

Table S49. Cartesian Coordinates and compliance matrix for $Ir(CO)_6^{3+}$ (MN15/def2-TZVPD)

C	0.000000	0.000000	2.056354
O	0.000000	0.000000	3.167043
C	-0.000000	-2.056354	-0.000000
O	-0.000000	-3.167043	-0.000000
C	-0.000000	0.000000	-2.056354
O	-0.000000	0.000000	-3.167043
C	-2.056354	0.000000	0.000000
O	-3.167043	0.000000	0.000000
C	2.056354	-0.000000	-0.000000
O	3.167043	-0.000000	-0.000000
C	0.000000	2.056354	-0.000000
O	0.000000	3.167043	-0.000000
Ir	0.000000	0.000000	-0.000000

	STRE(C,O)	STRE(C,Ir)
STRE(C,O)	0.044	
STRE(C,Ir)	-0.005	0.536

Table S50. Cartesian Coordinates and compliance matrix for $Ir(CO)_6^{3+}$ (ωB97X-D/def2-TZVPD)

C	0.000000	0.000000	2.061805
O	0.000000	0.000000	3.168041
C	-0.000000	-2.061805	-0.000000
O	-0.000000	-3.168041	-0.000000
C	-0.000000	0.000000	-2.061805
O	-0.000000	0.000000	-3.168041
C	-2.061805	0.000000	0.000000
O	-3.168041	0.000000	0.000000
C	2.061805	-0.000000	-0.000000
O	3.168041	-0.000000	-0.000000
C	0.000000	2.061805	-0.000000
O	0.000000	3.168041	-0.000000
Ir	0.000000	-0.000000	-0.000000

	STRE(C,O)	STRE(C,Ir)
STRE(C,O)	0.044	
STRE(C,Ir)	-0.007	0.677

Corresponding Author

* Joerg.Grunenberg@tu-braunschweig.de

V. References

[S1]: Gaussian 16, Revision C.01, M. J. Frisch, G. W. Trucks, H. B. Schlegel, G. E. Scuseria, M. A. Robb, J. R. Cheeseman, G. Scalmani, V. Barone, G. A. Petersson, H. Nakatsuji, X. Li, M. Caricato, A. V. Marenich, J. Bloino, B. G. Janesko, R. Gomperts, B. Mennucci, H. P. Hratchian, J. V. Ortiz, A. F. Izmaylov, J. L. Sonnenberg, D. Williams-Young, F. Ding, F. Lipparini, F. Egidi, J. Goings, B. Peng, A. Petrone, T. Henderson, D. Ranasinghe, V. G. Zakrzewski, J. Gao, N. Rega, G. Zheng, W. Liang, M. Hada, M. Ehara, K. Toyota, R. Fukuda, J. Hasegawa, M. Ishida, T. Nakajima, Y. Honda, O. Kitao, H. Nakai, T. Vreven, K. Throssell, J. A. Montgomery, Jr., J. E. Peralta, F. Ogliaro, M. J. Bearpark, J. J. Heyd, E. N. Brothers, K. N. Kudin, V. N. Staroverov, T. A. Keith, R. Kobayashi, J. Normand, K. Raghavachari, A. P. Rendell, J. C. Burant, S. S. Iyengar, J. Tomasi, M. Cossi, J. M. Millam, M. Klene, C. Adamo, R. Cammi, J. W. Ochterski, R. L. Martin, K. Morokuma, O. Farkas, J. B. Foresman, and D. J. Fox, Gaussian, Inc., Wallingford CT, 2016.

[S2]: GaussView, Version 6.1, Roy Dennington, Todd A. Keith, and John M. Millam, Semichem Inc., Shawnee Mission, KS, 2016.

[S3]: F. Weigend, R. Ahlrichs, *Phys. Chem. Chem. Phys.* **2005**, *7*, 3297.

[S4]: D. Rappoport, F. Furche, *J. Chem. Phys.* **2010**, *133*, 134105.

[S5]: D. Andrae, U. Häußermann, U. Dolg, H. Stoll, H. Preuß, *Theor. Chim. Acta* **1990**, *77*, 123-141.

[S6]: B. P. Pritchard, D. Altarawy, B. Didier, T. D. Gibson, T. L. Windus. *J. Chem. Inf. Model.* **2019**, *59*, 4814-4820.

[S7]: D. Feller, *J. Comput. Chem.* **1996**, *17*, 1571-1586.

[S8]: K. L. Schuchardt, B. T. Didier, T. Elsethagen, L. Sun, V. Gurumoorthi, C. Vidhya, J. Chase, J. Li, T. L. Windus, *J. Chem. Inf. Model.* **2019**, *59*, 4814-4820.

[S9]: K. Brandhorst, J. Grunenberg, *J. Chem. Phys.* **2010**, *132*, 184101-184107.

[S10]: K. Brandhorst, J. Grunenberg, *Chem. Soc. Rev.* **2008**, *37*, 1558-1567.
